# Association between parents’ socioeconomic conditions and nutritional status during childhood and the risk of cardiovascular disease in their adult offspring: an intergenerational study in south India

**DOI:** 10.1136/jech-2020-216261

**Published:** 2021-05-12

**Authors:** Poppy Alice Carson Mallinson, Bharati Kulkarni, Santhi Bhogadi, Sanjay Kinra

**Affiliations:** 1 Department of Non-Communicable Disease Epidemiology, London School of Hygiene & Tropical Medicine, London, UK; 2 Clinical Division, National Institute of Nutrition, Hyderabad, India; 3 Indian Institute of Public Health Hyderabad, Public Health Foundation of India, Hyderabad, India

**Keywords:** cardiovascular diseases, artherosclerosis, economics, lifecourse / childhood circumstances, growth

## Abstract

**Background:**

Some researchers have suggested that parents’ exposure to poor socioeconomic conditions during childhood can increase their offspring’s risk of cardiovascular disease, primarily through poor maternal nutrition and growth. However, epidemiological data on this association are limited. In an intergenerational cohort from rural India, we examined the association of parental childhood socioeconomic conditions and stature with offspring’s cardiovascular risk, hypothesising an inverse association between the two.

**Methods:**

We analysed data on 3175 adult offspring (aged 18–35 years, 58% men) and their parents from the third wave of the Andhra Pradesh Children and Parents’ Study (2010–12). We used multilevel linear regression to estimate the association of parents’ Standard of Living Index (SLI, an asset-based measure of socioeconomic conditions) in childhood, height and leg length with subclinical atherosclerosis and cardiovascular risk factors in their offspring.

**Results:**

In multivariable models adjusted for offspring’s socioeconomic conditions in childhood and adulthood, associations (beta coefficients and 95% CIs) of mother’s and father’s childhood SLI (per SD) were −0.00 mm (−0.01, 0.01) and 0.01 mm (−0.00, 0.02) for carotid intima media thickness, −0.17 mm Hg (−0.61, 0.27) and −0.30 mm Hg (−0.78, 0.20) for systolic blood pressure, −0.43 mg/dL (−2.00, 1.15) and −1.07 mg/dL (−2.79, 0.65) for total cholesterol and −0.00mU/L (−0.04, 0.03) and 0.01mU/L (−0.03, 0.04) for log fasting insulin. Results were of similar magnitude for parental height and leg length.

**Conclusions:**

Our findings do not support an inverse association between parental childhood socioeconomic conditions or stature and offspring’s risk of cardiovascular disease. Intergenerational socioeconomic influences on cardiovascular risk may be of limited public health significance for this setting.

## Introduction

There is an excess burden of premature cardiovascular mortality in South Asia, but the reasons for this are unclear.^1^ Socioeconomic conditions across the life course are an established determinant of an individual’s risk of cardiovascular disease.^2 3^ However, some recent evidence suggests that socioeconomic conditions experienced by an individual’s parents before their conception may also determine their risk of cardiovascular disease, independent of the individual’s own socioeconomic conditions.^4 5^ Several mechanisms have been proposed to explain the intergenerational effects of adverse socioeconomic conditions. The most notable of these is the influence of maternal childhood undernutrition on her own growth and consequently the growth of her offspring, which has been linked to future risk of cardiovascular disease.^6 7^ This mechanism could be important in South Asia where intergenerational poverty and undernutrition have been prevalent until recently.^8^ If true, this could suggest the need to consider childhood socioeconomic and/or nutritional interventions to control the cardiovascular disease epidemic in this region.

Despite the potential importance of the hypothesis, limited evidence supports the cardiovascular effects of intergenerational poverty or undernutrition. In studies from Sweden and the USA, socioeconomic position of the grandparents (as a marker of parental childhood socioeconomic conditions) was inversely associated with individual’s body mass index, although other cardiovascular risk factors were not examined.^9 10^ Evidence on the role of parents’ nutritional status in childhood for their offspring’s cardiovascular risk comes mostly from animal experiments.^11–13^ In the few epidemiological studies that have examined the association of parents’ stature (as a marker of their childhood nutrition) with cardiovascular disease and its risk factors in their offspring, findings have been inconsistent.^14–17^

The Andhra Pradesh Children and Parents’ Study (APCAPS) is an intergenerational cohort set in a rural area of south India, where poverty and undernutrition have been prevalent until recently.^18^ In the third wave of data collection, detailed information was collected on parents’ and offspring’s life course socioeconomic conditions and stature, along with a range of measures of cardiovascular risk including subclinical atherosclerosis, arterial stiffness and traditional cardiovascular risk factors. Our objective was to examine the association of parents’ socioeconomic conditions in childhood and stature with risk of cardiovascular disease in their offspring. We hypothesised an inverse association between parental childhood socioeconomic conditions and stature and their offspring’s risk of cardiovascular disease.

## Methods

This study is reported in accordance with the STROBE guidelines (see online supplemental table S1 for completed checklist). The APCAPS is set in 29 rural villages and towns ~50 km from the city of Hyderabad.^18^ APCAPS began as the long-term follow-up of offspring from a pregnancy nutrition trial conducted in 1987–1990 and was later expanded to cover family members of traced trial participants. In the present study, we used cross-sectional data from the third wave of data collection conducted in 2010–2012, in which 6944 of 10 213 (68%) invited family members attended a clinical examination. The index children who did not participate in the third wave were more likely to be women and have higher socioeconomic position, compared with previous waves, which was expected as these groups are more likely to leave their native villages for marriage or study/work, respectively.^19^ Adult offspring (aged 18–35 years) with at least one parent who was also examined were included in the analysis.

10.1136/jech-2020-216261.supp1 Supplementary data

### Clinical assessment

We collected social and demographic information using standard questions. These included a subset of questions from the Standard of Living Index (SLI), which is an asset-based scale developed to measure household wealth in India.^20^ To assess socioeconomic conditions during childhood, we asked participants to recall their household’s assets at age 10–12 years. We only asked about items expected to exhibit meaningful variation in this study setting (19 of out 29 original questions). These items were housing material, toilet facilities, lighting source, drinking water source, fuel source, whether they had a separate kitchen, and household possession of agricultural land, television, radio, clock, bicycle, motorcycle, car, telephone, refrigerator, water pump, cart, thresher and tractor. We derived the index by applying the recommended weights to the available household asset questions, then summing across all items to give a total score for each individual.^20^ A higher score indicates greater material affluence. Household-level measures of socioeconomic conditions are particularly appropriate for use in India, where joint family structures render individual-level measures (such as occupation and education) less informative. We assessed socioeconomic conditions in adulthood by current SLI (as above) as well as by occupational status (categorised as low—unemployed/unskilled manual labour; medium—skilled manual labour; high—nonmanual/professional or other—student/housewife/retired), in order to capture multiple dimensions that might be relevant to the risk of cardiovascular disease.^21^

Anthropometric measurements (standing height, sitting height, weight and waist circumference) were taken two times using standard equipment. The mean of the two readings was used for analyses. We examined both height and leg length as markers of parent’s nutritional status in childhood; height as it is more commonly used in the literature and leg length as it is the component of height thought to more specifically reflect late childhood nutrition and growth.^22 23^ Leg length was calculated as (standing height—(sitting height—stool height)). Body mass index was calculated as (weight in kilograms÷(height in metres^2^)).

Systolic and diastolic blood pressure were measured at the right upper arm in the sitting position using a validated oscillometric device (Omron M5-I model). Participants were asked to rest for 5 min before three readings were taken, each 1 min apart. The mean of the final two readings was used for analysis.

Venous blood samples were drawn after a minimum of 8 hours fasting and centrifuged immediately. Assays for fasting glucose were conducted locally on the same day as sampling using an enzymatic method. For all other assays, samples were transported in batches to our central laboratories for analysis. Total and high-density lipoprotein (HDL) cholesterol and triglycerides were estimated using enzymatic colorimetric methods and fasting insulin by radioimmunoassay. High-sensitivity C reactive protein (CRP) was measured using a particle-enhanced immunoturbidimetry method. Quality of assays was assured by internal duplicates and regular external standards.

A subsample of participants agreed to attend an additional clinic in Hyderabad for examination of subclinical measures of cardiovascular disease. Here, we measured intima-media thickness of the right common carotid artery, a marker of subclinical atherosclerosis, using B-mode ultrasonography (Ethiroli Tiny-16a, Surabi Biomedical Instrumentation, Coimbatore, India). A semiautomated software (AtheroEdge) was used to derive the mean of the measurements. We measured pulse wave velocity and augmentation index, markers of arterial stiffness, in the supine position using a Vicorder device (Skidmore Medical Limited, Bristol, UK). Body composition was measured by whole-body dual X-ray absorption scan (Hologic models Discovery A or 4500W) and standard Hologic software was used to derive abdominal fat mass.

### Data analysis

The following cardiovascular risk factors were considered in the analysis: body mass index, waist circumference, systolic blood pressure, diastolic blood pressure and fasting total cholesterol, HDL cholesterol, triglycerides, glucose, insulin and CRP, and in a subsample, carotid intima-media thickness, pulse wave velocity, augmentation index and abdominal fat mass. Before analysis, we checked distributions of outcome variables and excluded extreme outliers (defined as those judged to have implausible values after inspection of the data). Skewed variables (HDL cholesterol, triglycerides, glucose, insulin, CRP and abdominal fat mass) were log transformed to improve the normality of residuals.

To estimate the association of parental SLI in childhood, height and leg length with offspring’s cardiovascular risk factors, we used multilevel linear regression. We included a random intercept term at the family level to account for the potential correlation of cardiovascular risk factors between siblings. After first checking for evidence of nonlinearity in their association with offspring’s cardiovascular risk factors by testing whether addition of a quadratic term improved model fit, we used parental SLI in childhood, height and leg length as linear exposures. We fitted separate models for maternal and paternal exposures because a number of participants did not have data on both parents. Age and sex are strongly associated with cardiovascular risk factors, so we included both as covariates in all models. We included linear and quadratic terms for age to account for its nonlinear association with cardiovascular risk factors. Because socioeconomic conditions track strongly across generations, and adult and (to a lesser extent) childhood socioeconomic conditions are well-established determinants of cardiovascular disease risk, we present all models unadjusted and adjusted for indicators of offspring’s childhood and adult socioeconomic conditions. We conducted additional analyses stratified by sex of the offspring, as experimental studies in rodents indicate that associations between parental early life nutrition and offspring’s cardiovascular disease risk may be sex specific.^12 13^ We restricted all analyses to participants with complete outcome and exposure data, as data were missing for a relatively small number of participants. Power calculations indicated that with data on over 1000 offspring for all outcomes, we would have more than 80% power to detect even small effect sizes of <0.1 SD per SD change in exposure. We performed analysis using Stata V.16.^24^

To mitigate risk of type 1 error (ie, false positives) due to performing multiple statistical tests, we also report which p values are significant at a false discovery rate of 5% using the Benjamini-Hochberg method.^25^ For this, we considered all models with the same exposure as part of the same family of tests (ie, 14 tests per family).

Ethical approval for APCAPS was obtained from local review boards as well as the London School of Hygiene and Tropical Medicine. All participants provided written informed consent (or witnessed thumbprint if illiterate).

## Results

We analysed data on 3175 young adult offspring from 1434 families. The mean age of the offspring was 24 years (SD 4), and 58% were men (table 1). Data on the mother were available for 3016 offspring (95%), and data on the father were available for 2457 offspring (77%). Since most offspring (86%) had at least one sibling also in the study, the number of unique parents contributing data to the analysis was 2489 (1368 mothers and 1121 fathers). The mean age of mothers and fathers, respectively, was 47 years (SD 6) and 54 years (SD 7). Completeness of outcome data varied from 95% to 99%. Offspring with complete data on maternal exposures had similar sociodemographic profile to offspring with incomplete maternal data (online supplemental table S2). For paternal exposures, offspring with complete data were more likely to be men, younger and have higher SLI. A subsample of 1519 (48%) offspring attended the additional clinic for subclinical cardiovascular measurements. Compared with the overall sample, attendees were disproportionately men and likely to work in unskilled professions or be unemployed (to be expected since the clinic visit took most of a day). Among those who attended, outcome data were 88%–99% complete.

**Table 1:** Description of the study sample, Andhra Pradesh Children and Parents’ Study, 2010–2012

Offspring characteristic (N=3175)	% complete	N (%)/mean (SD)/median (IQR)
Age (years)		
Sex	>99%	24.4 (3.8)
Male	>99%	1842 (58%)
Female		1326 (42%)
Childhood SLI (out of 67)	>99%	15.9 (7.7)
Height (cm) (men/women)	>99%	167 (6.3) / 153 (5.7)
Leg length (cm) (men/women)	>99%	80.9 (4.1) / 74.7 (3.7)
Adult SLI (out of 67)	>99%	30.0 (8.5)
Adult occupation		
Unskilled labour or unemployed	>99%	803 (25%)
Student, retired or housewife		1233 (39%)
Semiskilled labour		331 (10%)
Skilled labour		522 (16%)
Professional		278 (9%)
Mother’s		
Age (years)	95%	46.9 (6.0)
Childhood SLI (out of 67)	95%	8.9 (4.4)
Height (cm)	95%	151 (5.4)
Leg length (cm)	95%	73.8 (3.6)
Father’s		
Age (years)	77%	54.2 (6.7)
Childhood SLI (out of 67)	77%	7.7 (3.9)
Height (cm)	77%	162 (6.3)
Leg length (cm)	77%	80.0 (4.1)
Cardiovascular risk factors		
Systolic blood pressure, mm Hg	>99%	115 (11.1)
Diastolic blood pressure, mm Hg	>99%	75 (10.7)
Total cholesterol, mg/dL	97%	157 (36)
HDL cholesterol, mg/dL	96%	41 (34, 49)
Triglycerides, mg/dL	96%	94 (70, 135)
Fasting glucose, mmol/dL	96%	5.0 (4.6, 5.3)
Fasting insulin, mU/L	95%	6.0 (3.9, 9.2)
C reactive protein, mg/L	96%	0.77 (0.35, 2.05)
Body mass index, kg/m^2^	>99%	20.6 (3.6)
Waist circumference, mm	>99%	70.7 (9.8)
Carotid intima-media thickness, mm	46% (97%)*	0.65 (0.17)
Pulse wave velocity, m/s	45% (94%)*	6.1 (0.8)
Augmentation index, %	42% (88%)*	15.6 (7.9)
Abdominal fat mass, kg	47% (99%)*	1.0 (0.6, 1.6)

*% of those who attended the additional clinic for subclinical cardiovascular measures.

HDL, high-density lipoprotein; SLI, Standard of Living Index.

Socioeconomic conditions were poorer in the parents’ childhood than for the offspring, as expected. Mean SLI score was 8/67 (SD 4) in parents’ childhood, 16/67 (SD 8) in offspring’s childhood and 30/67 (SD 9) for offspring currently. Parents had shorter height and leg length than their offspring (difference of 3.5 cm (SD 6) and 1.0 cm (SD 4), respectively, for both sexes). Among the offspring, prevalence of overweight and obesity (BMI ≥23 kg/m^2^) was 23%, while 37% had hypertension or prehypertension (blood pressure ≥120/80 mm Hg) and 4% had impaired fasting glucose (≥6.1 mmol/L).

**Figure 1:**
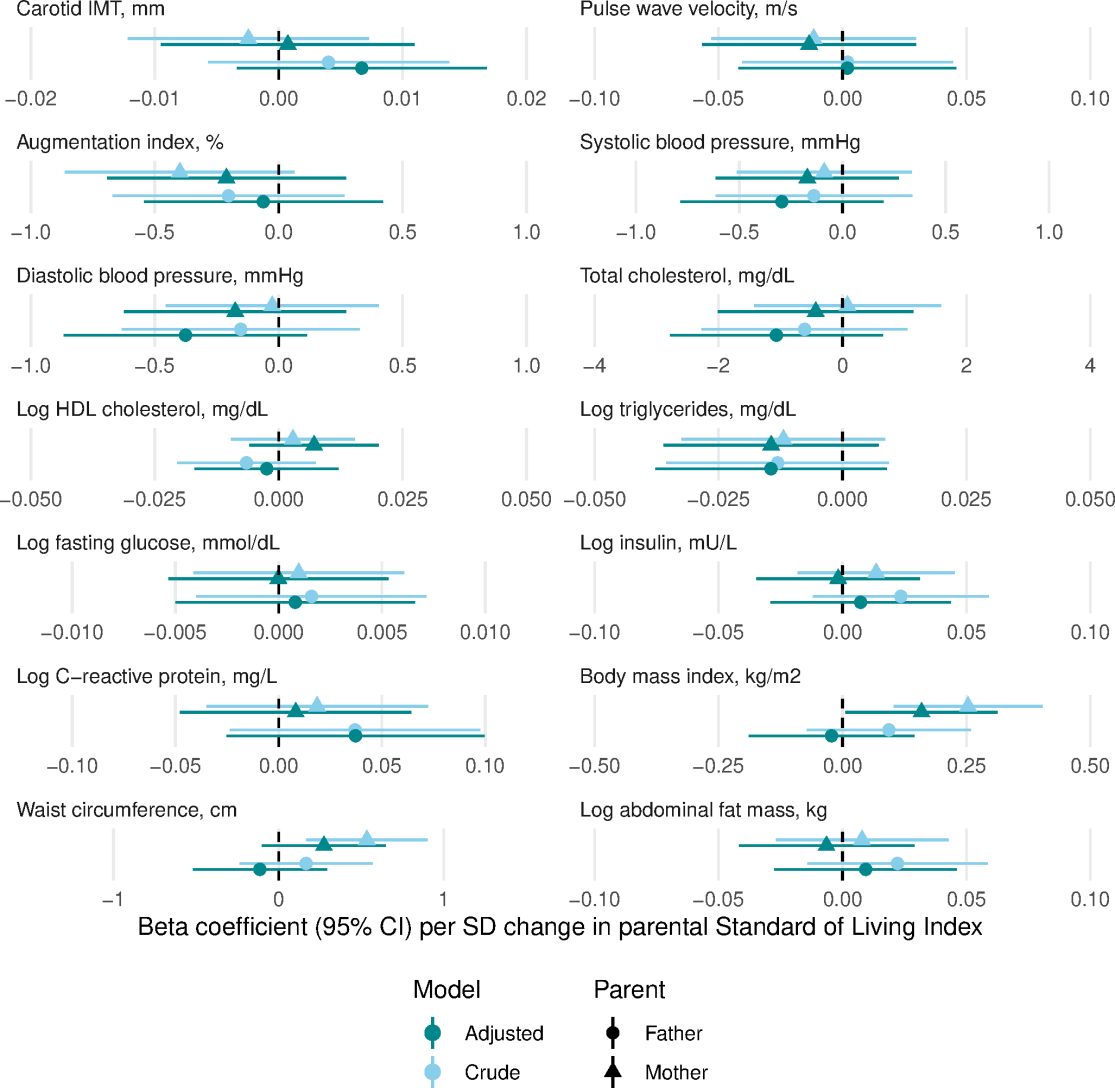
Linear regression beta coefficients for the association between parental childhood Standard of Living Index (SLI) and cardiovascular risk of the offspring in the Andhra Pradesh Children and Parents’ Study, 2010–2012. Crude models are adjusted for offspring’s age and sex; adjusted models are additionally adjusted for offspring’s childhood SLI (linear), adult SLI (linear) and adult occupation (categorical). No adjusted associations were significant after accounting for multiple testing (using Benjamini Hochberg method with 5% false discovery rate). IMT, intima media thickness; HDL, high-density lipoprotein.

**Figure 2:**
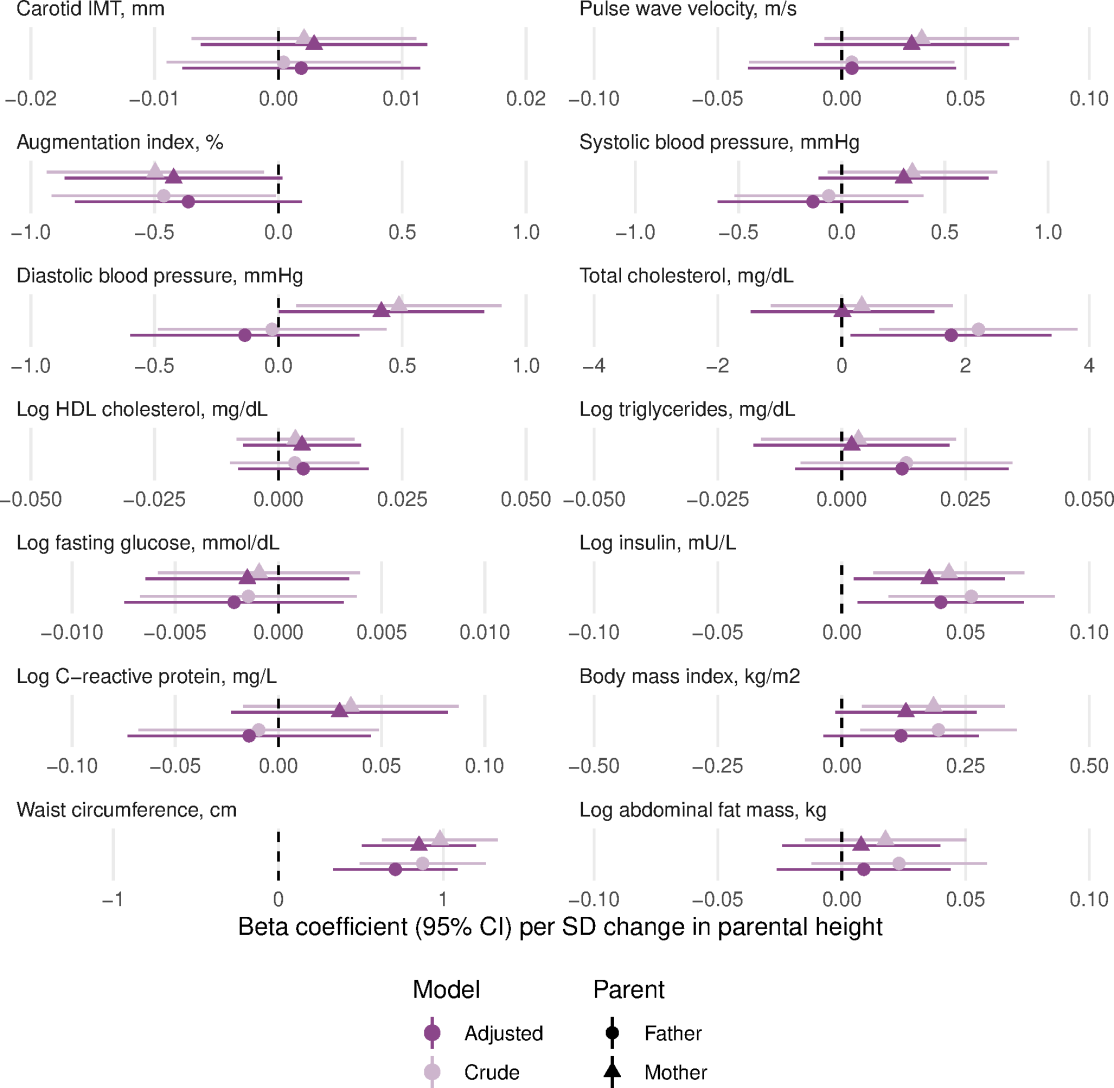
Linear regression beta coefficients for the association between parental height and cardiovascular risk of the offspring in the Andhra Pradesh Children and Parents’ Study, 2010–2012. Crude models are adjusted for offspring’s age and sex; adjusted models are additionally adjusted for offspring’s childhood SLI (linear), adult SLI (linear) and adult occupation (categorical). After adjustment, only the associations of mother’s and father’s height with offspring’s waist circumference were significant after accounting for multiple testing (using Benjamini Hochberg method with 5% false discovery rate). IMT, intima media thickness; HDL, high-density lipoprotein; SLI, Standard of Living Index.

**Figure 3:**
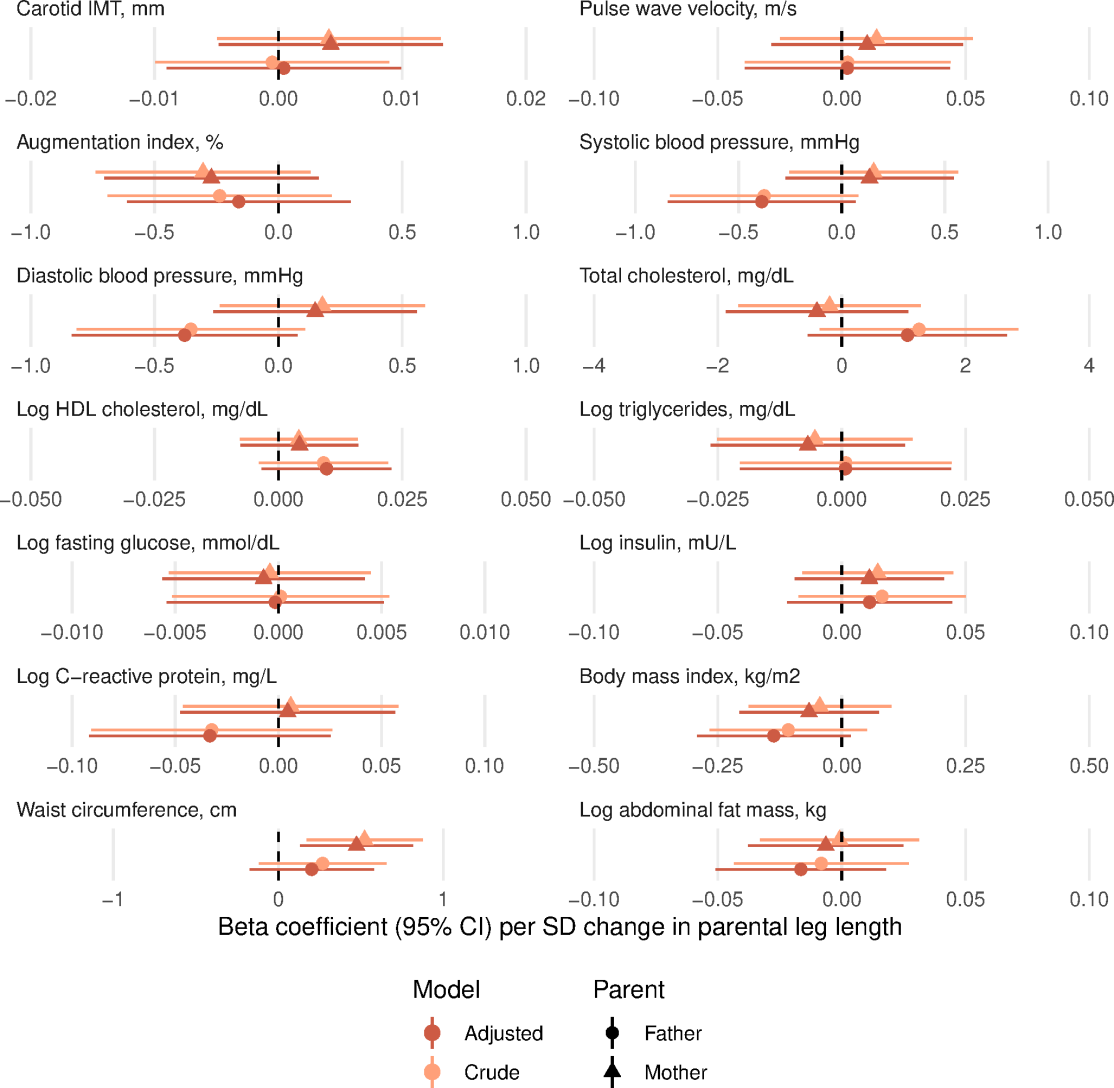
Linear regression beta coefficients for the association between parental leg length and cardiovascular risk of the offspring in the Andhra Pradesh Children and Parents’ Study, 2010–2012. Crude models are adjusted for offspring’s age and sex; adjusted models are additionally adjusted for offspring’s childhood SLI (linear), adult SLI (linear) and adult occupation (categorical). No adjusted associations were significant after accounting for multiple testing (using Benjamini Hochberg method with 5% false discovery rate). IMT, intima media thickness; HDL, high-density lipoprotein; SLI, Standard of Living Index.

In age-adjusted and sex-adjusted models, maternal childhood SLI was positively associated with offspring’s body mass index and waist circumference, but not with other risk factors, while paternal childhood SLI was not associated with any of the offspring’s cardiovascular risk factors (figure 1 and online supplemental table S3 and table S4). Maternal and paternal height were positively associated with offspring’s fasting insulin and waist circumference, with paternal height additionally associated with offspring’s total cholesterol (figure 2 and online supplemental tables S5 and S6). Maternal leg length was positively associated with offspring’s waist circumference only, while paternal leg length was not associated with any of the offspring’s cardiovascular risk factors (figure 3 and online supplemental table S7 and table S8). In the final models also adjusting for offspring’s childhood and adult socioeconomic conditions, only maternal and paternal height remained positively associated with offspring’s waist circumference. No other associations remained for any parental exposures.

We found no evidence that associations varied by sex of the offspring for most outcomes, except for paternal childhood SLI with offspring’s systolic and diastolic blood pressure: in female offspring, there was an inverse association (beta −0.75 mm Hg (−1.39 to –0.11), p=0.021; and beta −1.03 mm Hg (−1.67 to –0.40), p=0.001, per SD increase in paternal childhood SLI, respectively, the latter of which remained significant when considering a false discovery rate of 5%), but in male offspring, there was no association (beta 0.14 mm Hg (−0.43, 0.71), p=0.6; and beta 0.24 mm Hg (−0.33, 0.81), p=0.4).

## Discussion

In this rural Indian setting, we found no clear evidence that parents’ socioeconomic conditions in childhood or stature were associated with subclinical atherosclerosis or cardiovascular risk factors in their offspring.

### Relation to the literature

As far as we are aware, few studies globally, and none from South Asia, have examined the association between parents’ socioeconomic conditions in childhood and risk of cardiovascular disease in their offspring. In particular, there is a lack of evidence for offspring outcomes other than obesity. In a cohort of young adults from a town in Sweden (N=~5000, 100% men), income of their grandparents (who were born between 1915 and 1929) was inversely associated with participants’ body mass index, even after adjusting for their parents’ income.^9^ In a nationally representative cohort from the USA (N=4648, response rate ~50%), educational level of the grandparents (born in ~1900–1940) was inversely associated with obesity in white but not black offspring, which persisted after adjustment for parents’ and offspring’s education.^10^ One possible reason for the discrepancy between findings from these studies and our own study is residual confounding by the offspring’s socioeconomic conditions in adulthood. The inverse associations observed in previous studies may reflect the well-established inverse association between socioeconomic conditions in adulthood and obesity in high-income countries, rather than an independent association with parental childhood conditions.^26^ Conversely, a true inverse association between parental childhood socioeconomic conditions and offspring obesity may have been masked in our analysis because of the positive association between adult socioeconomic conditions and obesity in India.^27^

Similarly, few studies have examined the association between parental stature (a marker of nutritional status and growth in childhood) and offspring’s risk of cardiovascular disease.^14–17^ These studies examined parental height only; to our knowledge, no previous studies have examined the association between parental leg length and offspring cardiovascular risk. In a family-based cohort from two towns in Scotland conducted in 1993–1994 (N=2306), maternal, but not paternal, height was inversely associated with prevalent coronary heart disease (diagnosed by ECG) in the offspring, which persisted after adjustment for parental socioeconomic conditions.^14^ However, in the same study, maternal height was positively associated with offspring’s waist circumference, and not associated with other cardiovascular risk factors. It is interesting that we also found offspring’s waist circumference, but not other measures of adiposity, to be positively associated with parental height, although this may reflect the fact that an individual’s waist circumference (unlike the other measures of adiposity) is correlated with their height. In analyses of a cohort of individuals born in a hospital in Finland between 1924 and 1933 (N=3302 men and 3447 women), maternal height was not associated with the offspring’s coronary heart disease mortality or hospitalisation.^16 17^ An analysis of birth cohort studies from four middle-income countries (Brazil, South Africa, Philippines and Guatemala, N=~10 000) found no evidence of association of maternal height with body mass index, blood pressure or plasma glucose in their offspring aged ~20–30 years.^15^ Although no data from low-income countries were included, it is notable that similar null findings were observed across cohorts ranging from urban and upper middle income (Brazil) to rural and lower middle income (Guatemala).

Our finding of an inverse association between paternal socioeconomic conditions in childhood and blood pressure in female but not male offspring is intriguing but is not consistent with previous studies.^14 15^ Confirmation in further studies is warranted.

### Strengths and limitations

Our study is among few to have examined intergenerational socioeconomic influences on cardiovascular health. We used data from a large population-based study in a rural area of India, where undernutrition, a key proposed mechanism of this association, has been prevalent until recently. We assessed cardiovascular risk in the offspring using a comprehensive range cardiovascular risk factors and markers of subclinical disease.

Some limitations must be acknowledged. First, we relied on parents’ recall of their childhood socioeconomic conditions, which may be prone to measurement error and thus have caused associations to be attenuated towards the null. However, in a repeat questionnaire administered to a 5% subsample, reliability of these responses was found to be high (over 80% correlation between the two measures), suggesting that measurement error may not have been a substantial problem. Second, we cannot exclude residual confounding by offspring socioeconomic factors. Although we adjusted for multiple indicators of offspring’s socioeconomic conditions, this construct is inherently difficult to capture in epidemiological studies. At the same time, there is a risk that including offspring’s socioeconomic conditions in our models could have introduced collider stratification bias if there were unmeasured causes of both offspring socioeconomic conditions and the exposure or outcome. However, as both of these potential biases would tend to bias associations away from the null, they are unlikely to have substantially affected our main conclusions (of no association). Third, although minimal data were missing for most exposures and outcomes (less than 5%), data on paternal exposures were missing for 23% of participants, which could have introduced bias in the analyses of paternal exposures. We opted not to use multiple imputation because we included several sociodemographic variables that might predict missingness in our final models (such as age, sex and occupation) and assumed that beyond these, missingness would not depend strongly on the outcome (as participants were largely unaware of their outcome status); in such a situation multiple imputation offers little additional benefit.^28^ Fourth, although our study setting is in many ways typical of rural areas within India and South Asia, caution should be exercised when generalising these findings to urban areas or other countries.

## Conclusions

We had hypothesised that poor parental socioeconomic conditions in childhood, and associated undernutrition and stunted growth, leads to raised cardiovascular risk in the offspring. Given the high levels of poverty and stunting in South Asia, we thought this might contribute substantially to the excess premature cardiovascular mortality in South Asia. Contrary to these hypotheses, our findings suggest that intergenerational influences of poverty and undernutrition may be less important for cardiovascular disease risk than has been proposed based on findings from animal experiments and limited epidemiological data from high-income countries.^4 5^ Socioeconomic factors acting within an individual’s lifetime may be more fruitful targets for interventions to reduce cardiovascular mortality and its inequalities in South Asia. Further studies with prospective measures of parental childhood conditions and longer term follow-up are needed to confirm this.

**What is already known on this subject**

- Researchers have hypothesised that adverse socioeconomic conditions in childhood, and associated undernutrition and poor growth, may increase risk of cardiovascular disease in the next generation.
- However, epidemiological evidence is limited, especially in settings where intergenerational poverty and undernutrition remain prevalent.

**What this study adds**

- In a rural setting in India, we found no clear evidence to support an inverse association between parents’ socioeconomic conditions in childhood or stature with levels of subclinical atherosclerosis or cardiovascular risk factors in their adult offspring.
- These findings suggest that intergenerational socioeconomic influences on cardiovascular risk may be of limited public health significance for this setting.

## Data Availability

Data are available upon reasonable request. Data cannot be made fully available without restriction because of stipulations in our original participant consent forms. Data are available to researchers through completion of a collaborator form (https://apcaps.lshtm.ac.uk/apply-to-collaborate/).
