# Supplementary material for: Association between parents’ socioeconomic conditions and nutritional status during childhood and the risk of cardiovascular disease in their adult offspring: an intergenerational study in south India
Source: J Epidemiol Community Health. 2021 May 12;75(11):1091–7. doi: 10.1136/jech-2020-216261 (PMC8515111; doi:10.1136/jech-2020-216261)
Supplement: Supplementary data [file jech-2020-216261supp001.pdf]

Supplementary Material

Table S1: STROBE Statement—Checklist of items that should be included in reports of *cross-sectional studies*

	Item No	Recommendation	Page
**Title and abstract**	1	(*a*) Indicate the study's design with a commonly used term in the title or the abstract	1, 2
		(*b*) Provide in the abstract an informative and balanced summary of what was done and what was found	2
**Introduction**			
Background/ rationale	2	Explain the scientific background and rationale for the investigation being reported	4
Objectives	3	State specific objectives, including any prespecified hypotheses	5
**Methods**			
Study design	4	Present key elements of study design early in the paper	5
Setting	5	Describe the setting, locations, and relevant dates, including periods of recruitment, exposure, follow-up, and data collection	5
Participants	6	(*a*) Give the eligibility criteria, and the sources and methods of selection of participants	5
Variables	7	Clearly define all outcomes, exposures, predictors, potential confounders, and effect modifiers. Give diagnostic criteria, if applicable	7, 8
Data sources/ measurement	8*	For each variable of interest, give sources of data and details of methods of assessment (measurement). Describe comparability of assessment methods if there is more than one group	5, 6, 7
Bias	9	Describe any efforts to address potential sources of bias	7, 8
Study size	10	Explain how the study size was arrived at	5, 8
Quantitative variables	11	Explain how quantitative variables were handled in the analyses. If applicable, describe which groupings were chosen and why	6, 7, 8
Statistical methods	12	(*a*) Describe all statistical methods, including those used to control for confounding	7, 8
		(*b*) Describe any methods used to examine subgroups and interactions	8
		(*c*) Explain how missing data were addressed	8
		(*d*) If applicable, describe analytical methods taking account of sampling strategy	NA
		(*e*) Describe any sensitivity analyses	8
**Results**			
Participants	13*	(a) Report numbers of individuals at each stage of study—eg numbers potentially eligible, examined for eligibility, confirmed eligible, included in the study, completing follow-up, and analysed	5, 9
		(b) Give reasons for non-participation at each stage	5, 9
		(c) Consider use of a flow diagram	NA
Descriptive data	14*	(a) Give characteristics of study participants (eg demographic, clinical, social) and information on exposures and potential confounders	9

Mallinson PAC, *et al. J Epidemiol Community Health* 2021; 75:1091–1097. doi: 10.1136/jech-2020-216261

		(b) Indicate number of participants with missing data for each variable of interest	9, Table 1
Outcome data	15*	Report numbers of outcome events or summary measures	11, Table 1
Main results	16	(*a*) Give unadjusted estimates and, if applicable, confounder-adjusted estimates and their precision (eg, 95% confidence interval). Make clear which confounders were adjusted for and why they were included	11, Figures 1-3
		(*b*) Report category boundaries when continuous variables were categorized	NA
		(*c*) If relevant, consider translating estimates of relative risk into absolute risk for a meaningful time period	NA
Other analyses	17	Report other analyses done—eg analyses of subgroups and interactions, and sensitivity analyses	11, 12
**Discussion**			
Key results	18	Summarise key results with reference to study objectives	12
Limitations	19	Discuss limitations of the study, taking into account sources of potential bias or imprecision. Discuss both direction and magnitude of any potential bias	13, 14
Interpretation	20	Give a cautious overall interpretation of results considering objectives, limitations, multiplicity of analyses, results from similar studies, and other relevant evidence	12, 13, 17
Generalisability	21	Discuss the generalisability (external validity) of the study results	14
**Other information**			
Funding	22	Give the source of funding and the role of the funders for the present study and, if applicable, for the original study on which the present article is based	16

*Give information separately for exposed and unexposed groups.

Note: An Explanation and Elaboration article discusses each checklist item and gives methodological background and published examples of transparent reporting. The STROBE checklist is best used in conjunction with this article (freely available on the Web sites of PLoS Medicine at http://www.plosmedicine.org/, Annals of Internal Medicine at http://www.annals.org/, and Epidemiology at http://www.epidem.com/). Information on the STROBE Initiative is available at www.strobe-statement.org.

Table S2: Difference between participants with complete vs incomplete data, Andhra Pradesh Children and Parents' Study (APCAPS), 2010-2012.

Socio-demographic characteristics		Maternal exposures			Paternal exposures			Additional clinic for subclinical cardiovascular measures		
		Complete data (n=2795)	Incomplete data* (n=359)	p-value	Complete data (n=2272)	Incomplete data* (n=882)	p-value	Complete data (n=1286)	Incomplete data (n=1868)	p-value
Age		24.3 (3.8)	24.8 (3.9)	0.02	24.2 (3.7)	24.9 (4.0)	<0.001	24.3 (4.0)	24.4 (3.7)	0.18
Sex	Male	1641 (58%)	193 (54%)	0.07	1351 (59%)	483 (55%)	0.02	786 (61%)	1048 (56%)	0.01
	Female	1154 (41%)	166 (46%)		921 (41%)	399 (45%)		500 (39%)	820 (44%)	
Childhood Standard of Living Index		15.9 (7.6)	15.9 (8.1)	0.98	16.2 (7.6)	15.0 (7.6)	<0.001	16.0 (7.7)	15.8 (7.6)	0.38
Adult Standard of Living Index		29.9 (8.4)	30.4 (9.5)	0.28	30.4 (8.4)	29.0 (8.7)	<0.001	29.9 (8.3)	30.0 (8.7)	0.72
Adult occupation	Unskilled labour or unemployed	699 (25%	102 (28%)	0.28	558 (25%)	243 (28%)	0.24	367 (29%)	434 (23%)	0.001
	Student, retired or housewife	1092 (39%)	135 (38%)		900 (40%)	327 (37%)		493 (38%)	734 (39%)	
	Semi-skilled labour	286 (10%)	44 (12%)		242 (11%)	88 (10%)		143 (11%)	187 (10%)	
	Skilled labour	468 (17%)	53 (15%)		366 (16%)	155 (18%)		190 (15%)	331 (18%)	
	Professional	250 (9%)	25 (7%)		206 (9%)	69 (8%)		93 (7%)	182 (10%)	

*Incomplete data includes participants with incomplete data for any of the main cardiovascular risk factors. All counts exclude 21 participants with incomplete data on socio-demographic characteristics.

Mallinson PAC, *et al. J Epidemiol Community Health* 2021; 75:1091–1097. doi: 10.1136/jech-2020-216261

Table S3: Association between mother's childhood standard of living index (SLI) and cardiovascular risk of the offspring in the Andhra Pradesh Children and Parents' Study (APCAPS), 2010-2012.

Cardiovascular risk factor	Model 1: Age- and sex-adjusted					Model 2: model 1 + offspring's childhood and adult socioeconomic conditions[1]				
	N	β	Lower CI	Upper CI	p-value	N	β	Lower CI	Upper CI	p-value
Carotid IMT, mm	1317	-0.002	-0.012	0.007	0.622	1312	0.001	-0.009	0.011	0.887
Pulse wave velocity, m/s	1310	-0.012	-0.053	0.030	0.581	1307	-0.013	-0.057	0.030	0.541
Augmentation index, %	1230	-0.399	-0.862	0.064	0.091	1227	-0.211	-0.693	0.271	0.391
Systolic blood pressure, mmHg	2751	-0.088	-0.512	0.335	0.682	2728	-0.170	-0.613	0.273	0.452
Diastolic blood pressure, mmHg	2751	-0.026	-0.456	0.404	0.905	2728	-0.175	-0.623	0.272	0.443
Total cholesterol, mg/dL	2666	0.080	-1.427	1.587	0.917	2645	-0.431	-2.009	1.146	0.592
Log HDL cholesterol, mg/dL	2659	0.003	-0.010	0.015	0.652	2638	0.007	-0.006	0.020	0.283
Log triglycerides, mg/dL	2650	-0.012	-0.032	0.009	0.256	2629	-0.014	-0.036	0.007	0.193
Log fasting glucose, mmol/dL	2657	0.001	-0.004	0.006	0.710	2637	0.000	-0.005	0.005	0.996
Log fasting insulin, mU/L	2624	0.014	-0.018	0.045	0.400	2603	-0.002	-0.035	0.031	0.918
Log C-reactive protein, mg/L	2660	0.019	-0.035	0.072	0.497	2639	0.008	-0.048	0.064	0.772
Body mass index, kg/m^2	2744	0.253	0.103	0.403	0.001*	2721	0.160	0.006	0.313	0.042
Waist circumference, mm	2737	0.534	0.166	0.901	0.004*	2714	0.274	-0.100	0.648	0.151
Log abdominal fat mass, kg	1344	0.008	-0.027	0.043	0.657	1339	-0.006	-0.042	0.029	0.721

IMT is intima media thickness; HDL is high-density lipoprotein. Beta coefficient represents the effect of a 1-SD change in mother's childhood SLI.

[1]Childhood SLI (linear), adult standard of living index (linear) and adult occupation (categorical)

*P-value significant after accounting for multiple testing (using Benjamini Hochberg method with 5% false discovery rate)

Table S4: Association between father's childhood standard of living index (SLI) and cardiovascular risk of the offspring in the Andhra Pradesh Children and Parents' Study (APCAPS), 2010-2012.

Cardiovascular risk factor	Model 1: Age- and sex-adjusted					Model 2: model 1 + offspring's childhood and adult socioeconomic conditions[1]				
	N	β	Lower CI	Upper CI	p-value	N	β	Lower CI	Upper CI	p-value
Carotid IMT, mm	1098	0.004	-0.006	0.014	0.418	1095	0.007	-0.003	0.017	0.192
Pulse wave velocity, m/s	1084	0.002	-0.040	0.045	0.924	1081	0.002	-0.042	0.046	0.930
Augmentation index, %	1019	-0.203	-0.670	0.265	0.395	1016	-0.062	-0.544	0.420	0.801
Systolic blood pressure, mmHg	2240	-0.138	-0.614	0.338	0.569	2226	-0.294	-0.784	0.197	0.241
Diastolic blood pressure, mmHg	2240	-0.153	-0.633	0.326	0.531	2226	-0.376	-0.867	0.115	0.133
Total cholesterol, mg/dL	2169	-0.611	-2.270	1.048	0.470	2157	-1.067	-2.786	0.651	0.224
Log HDL cholesterol, mg/dL	2166	-0.007	-0.021	0.007	0.362	2154	-0.002	-0.017	0.012	0.744
Log triglycerides, mg/dL	2158	-0.013	-0.036	0.009	0.253	2146	-0.014	-0.038	0.009	0.225
Log fasting glucose, mmol/dL	2166	0.002	-0.004	0.007	0.576	2154	0.001	-0.005	0.007	0.786
Log fasting insulin, mU/L	2138	0.024	-0.012	0.059	0.192	2126	0.007	-0.029	0.044	0.693
Log C-reactive protein, mg/L	2163	0.037	-0.024	0.097	0.231	2151	0.037	-0.025	0.100	0.242
Body mass index, kg/m^2	2233	0.094	-0.072	0.259	0.266	2219	-0.022	-0.189	0.145	0.795
Waist circumference, mm	2227	0.166	-0.237	0.569	0.420	2213	-0.114	-0.519	0.291	0.581
Log abdominal fat mass, kg	1110	0.022	-0.014	0.059	0.232	1107	0.009	-0.027	0.046	0.618

IMT is intima media thickness; HDL is high-density lipoprotein. Beta coefficient represents the effect of a 1-SD change in father's childhood SLI.

[1]Childhood SLI (linear), adult standard of living index (linear) and adult occupation (categorical)

*P-value significant after accounting for multiple testing (using Benjamini Hochberg method with 5% false discovery rate)

Table S5: Association between mother's height and cardiovascular risk of the offspring in the Andhra Pradesh Children and Parents' Study (APCAPS), 2010-2012.

Cardiovascular risk factor	Model 1: Age- and sex-adjusted					Model 2: model 1 + offspring's childhood and adult socioeconomic conditions[1]				
	N	β	Lower CI	Upper CI	p-value	N	β	Lower CI	Upper CI	p-value
Carotid IMT, mm	1396	0.002	-0.007	0.011	0.654	1391	0.003	-0.006	0.012	0.537
Pulse wave velocity, m/s	1358	0.032	-0.007	0.072	0.106	1353	0.028	-0.011	0.068	0.159
Augmentation index, %	1272	-0.498	-0.936	-0.060	0.026	1267	-0.423	-0.863	0.016	0.059
Systolic blood pressure, mmHg	3003	0.342	-0.068	0.753	0.102	2990	0.300	-0.111	0.711	0.153
Diastolic blood pressure, mmHg	3003	0.487	0.072	0.902	0.021	2990	0.416	0.002	0.830	0.049
Total cholesterol, mg/dL	2913	0.325	-1.146	1.796	0.665	2900	0.011	-1.472	1.493	0.989
Log HDL cholesterol, mg/dL	2906	0.003	-0.008	0.015	0.572	2893	0.005	-0.007	0.017	0.434
Log triglycerides, mg/dL	2896	0.003	-0.016	0.023	0.736	2884	0.002	-0.018	0.022	0.842
Log fasting glucose, mmol/dL	2903	-0.001	-0.006	0.004	0.708	2890	-0.002	-0.006	0.003	0.548
Log fasting insulin, mU/L	2871	0.043	0.013	0.074	0.005*	2858	0.035	0.005	0.066	0.022
Log C-reactive protein, mg/L	2906	0.035	-0.017	0.087	0.187	2893	0.030	-0.023	0.082	0.268
Body mass index, kg/m^2	2996	0.185	0.041	0.330	0.012	2983	0.130	-0.013	0.272	0.074
Waist circumference, mm	2988	0.979	0.629	1.329	<0.001*	2975	0.852	0.507	1.196	<0.001*
Log abdominal fat mass, kg	1428	0.018	-0.015	0.050	0.286	1423	0.008	-0.024	0.040	0.630

IMT is intima media thickness; HDL is high-density lipoprotein. Beta coefficient represents the effect of a 1-SD (5.4cm) change in mother's height.
[1]Childhood SLI (linear), adult standard of living index (linear) and adult occupation (categorical)
*P-value significant after accounting for multiple testing (using Benjamini Hochberg method with 5% false discovery rate)

Mallinson PAC, *et al. J Epidemiol Community Health* 2021; 75:1091–1097. doi: 10.1136/jech-2020-216261

Table S6: Association between father's height and cardiovascular risk of the offspring in the Andhra Pradesh Children and Parents' Study (APCAPS), 2010-2012.

Cardiovascular risk factor	Model 1: Age- and sex-adjusted					Model 2: model 1 + offspring's childhood and adult socioeconomic conditions[1]				
	N	β	Lower CI	Upper CI	p-value	N	β	Lower CI	Upper CI	p-value
Carotid IMT, mm	1165	0.000	-0.009	0.010	0.932	1160	0.002	-0.008	0.011	0.706
Pulse wave velocity, m/s	1129	0.004	-0.037	0.046	0.848	1124	0.004	-0.038	0.046	0.847
Augmentation index, %	1057	-0.463	-0.916	-0.011	0.045	1052	-0.364	-0.822	0.094	0.120
Systolic blood pressure, mmHg	2444	-0.063	-0.520	0.395	0.787	2433	-0.139	-0.600	0.321	0.553
Diastolic blood pressure, mmHg	2444	-0.025	-0.486	0.435	0.914	2433	-0.136	-0.597	0.326	0.565
Total cholesterol, mg/dL	2365	2.209	0.612	3.807	0.007*	2354	1.769	0.149	3.389	0.032
Log HDL cholesterol, mg/dL	2362	0.003	-0.010	0.016	0.618	2351	0.005	-0.008	0.018	0.455
Log triglycerides, mg/dL	2353	0.013	-0.008	0.034	0.231	2342	0.012	-0.009	0.034	0.266
Log fasting glucose, mmol/dL	2360	-0.001	-0.007	0.004	0.586	2349	-0.002	-0.007	0.003	0.428
Log fasting insulin, mU/L	2334	0.052	0.019	0.086	0.002*	2323	0.040	0.006	0.074	0.019
Log C-reactive protein, mg/L	2358	-0.010	-0.068	0.049	0.748	2347	-0.014	-0.073	0.045	0.637
Body mass index, kg/m^2	2436	0.195	0.037	0.353	0.015	2425	0.120	-0.037	0.276	0.134
Waist circumference, mm	2428	0.874	0.492	1.256	<0.001*	2417	0.708	0.331	1.086	<0.001*
Log abdominal fat mass, kg	1181	0.023	-0.012	0.059	0.200	1176	0.009	-0.026	0.044	0.618

IMT is intima media thickness; HDL is high-density lipoprotein. Beta coefficient represents the effect of a 1-SD (6.3cm) change in father's height.
[1]Childhood SLI (linear), adult standard of living index (linear) and adult occupation (categorical)
*P-value significant after accounting for multiple testing (using Benjamini Hochberg method with 5% false discovery rate)

Table S7: Association between mother's leg length and cardiovascular risk of the offspring in the Andhra Pradesh Children and Parents' Study (APCAPS), 2010-2012.

Cardiovascular risk factor	Model 1: Age- and sex-adjusted					Model 2: model 1 + offspring's childhood and adult socioeconomic conditions[1]				
	N	β	Lower CI	Upper CI	p-value	N	β	Lower CI	Upper CI	p-value
Carotid IMT, mm	1393	0.004	-0.005	0.013	0.376	1388	0.004	-0.005	0.013	0.358
Pulse wave velocity, m/s	1357	0.014	-0.025	0.053	0.477	1352	0.010	-0.028	0.049	0.603
Augmentation index, %	1271	-0.304	-0.737	0.129	0.168	1266	-0.271	-0.703	0.161	0.219
Systolic blood pressure, mmHg	2995	0.155	-0.254	0.564	0.458	2982	0.135	-0.273	0.542	0.517
Diastolic blood pressure, mmHg	2995	0.177	-0.236	0.591	0.400	2982	0.148	-0.262	0.559	0.479
Total cholesterol, mg/dL	2905	-0.196	-1.666	1.275	0.794	2892	-0.400	-1.874	1.074	0.595
Log HDL cholesterol, mg/dL	2898	0.004	-0.008	0.016	0.497	2885	0.004	-0.008	0.016	0.481
Log triglycerides, mg/dL	2888	-0.005	-0.025	0.014	0.590	2876	-0.007	-0.026	0.013	0.495
Log fasting glucose, mmol/dL	2895	0.000	-0.005	0.004	0.867	2882	-0.001	-0.006	0.004	0.773
Log fasting insulin, mU/L	2863	0.015	-0.016	0.045	0.349	2850	0.011	-0.019	0.041	0.469
Log C-reactive protein, mg/L	2898	0.006	-0.046	0.058	0.822	2885	0.004	-0.048	0.057	0.866
Body mass index, kg/m^2	2988	-0.044	-0.188	0.100	0.547	2975	-0.066	-0.207	0.075	0.359
Waist circumference, mm	2980	0.522	0.170	0.873	0.004*	2967	0.473	0.130	0.816	0.007
Log abdominal fat mass, kg	1425	-0.001	-0.033	0.031	0.957	1420	-0.006	-0.038	0.025	0.688

IMT is intima media thickness; HDL is high-density lipoprotein. Beta coefficient represents the effect of a 1-SD (3.6cm) change in mother's leg length.

[1]Childhood SLI (linear), adult standard of living index (linear) and adult occupation (categorical)

*P-value significant after accounting for multiple testing (using Benjamini Hochberg method with 5% false discovery rate)

Table S8: Association between father's leg length and cardiovascular risk of the offspring in the Andhra Pradesh Children and Parents' Study (APCAPS), 2010-2012.

Cardiovascular risk factor	Model 1: Age- and sex-adjusted					Model 2: model 1 + offspring's childhood and adult socioeconomic conditions[1]				
	N	β	Lower CI	Upper CI	p-value	N	β	Lower CI	Upper CI	p-value
Carotid IMT, mm	1165	-0.001	-0.010	0.009	0.917	1160	0.000	-0.009	0.010	0.928
Pulse wave velocity, m/s	1129	0.002	-0.039	0.044	0.910	1124	0.002	-0.039	0.044	0.914
Augmentation index, %	1057	-0.237	-0.689	0.214	0.303	1052	-0.160	-0.611	0.291	0.486
Systolic blood pressure, mmHg	2442	-0.376	-0.833	0.081	0.107	2431	-0.388	-0.842	0.067	0.095
Diastolic blood pressure, mmHg	2442	-0.354	-0.814	0.107	0.133	2431	-0.379	-0.836	0.078	0.104
Total cholesterol, mg/dL	2363	1.250	-0.354	2.854	0.127	2352	1.062	-0.546	2.670	0.195
Log HDL cholesterol, mg/dL	2360	0.009	-0.004	0.022	0.172	2349	0.010	-0.003	0.023	0.145
Log triglycerides, mg/dL	2351	0.001	-0.021	0.022	0.940	2340	0.001	-0.021	0.022	0.945
Log fasting glucose, mmol/dL	2358	0.000	-0.005	0.005	0.971	2347	0.000	-0.005	0.005	0.954
Log fasting insulin, mU/L	2332	0.016	-0.017	0.050	0.342	2321	0.011	-0.022	0.044	0.508
Log C-reactive protein, mg/L	2356	-0.032	-0.091	0.026	0.278	2345	-0.033	-0.092	0.025	0.265
Body mass index, kg/m^2	2434	-0.108	-0.266	0.051	0.182	2423	-0.137	-0.292	0.018	0.083
Waist circumference, mm	2426	0.268	-0.118	0.654	0.174	2415	0.201	-0.175	0.577	0.295
Log abdominal fat mass, kg	1181	-0.008	-0.044	0.027	0.647	1176	-0.016	-0.051	0.018	0.348

IMT is intima media thickness; HDL is high-density lipoprotein. Beta coefficient represents the effect of a 1-SD (4.1cm) change in father's leg length.
[1]Childhood SLI (linear), adult standard of living index (linear) and adult occupation (categorical)
*P-value significant after accounting for multiple testing (using Benjamini Hochberg method with 5% false discovery rate)
